# Expression of B-RAF V600E in Type II Pneumocytes Causes Abnormalities in Alveolar Formation, Airspace Enlargement and Tumor Formation in Mice

**DOI:** 10.1371/journal.pone.0029093

**Published:** 2011-12-14

**Authors:** Emanuele Zanucco, Rudolf Götz, Tamara Potapenko, Irene Carraretto, Semra Ceteci, Fatih Ceteci, Werner Seeger, Rajkumar Savai, Ulf R. Rapp

**Affiliations:** 1 Department of Molecular Biology, Max Planck Institute of Biochemistry, Martinsried, Germany; 2 Institute for Medical Radiation and Cell Research, University of Würzburg, Würzburg, Germany; 3 Department of Lung Development and Remodeling, Max Planck Institute for Heart and Lung Research, Bad Nauheim, Germany; Comprehensive Pneumology Center, Germany

## Abstract

Growth factor induced signaling cascades are key regulatory elements in tissue development, maintenance and regeneration. Perturbations of these cascades have severe consequences, leading to developmental disorders and neoplastic diseases. As a major function in signal transduction, activating mutations in RAF family kinases are the cause of human tumorigenesis, where B-RAF V600E has been identified as the prevalent mutant. In order to address the oncogenic function of B-RAF V600E, we have generated transgenic mice expressing the activated oncogene specifically in lung alveolar epithelial type II cells. Constitutive expression of B-RAF V600E caused abnormalities in alveolar epithelium formation that led to airspace enlargements. These lung lesions showed signs of tissue remodeling and were often associated with chronic inflammation and low incidence of lung tumors. The inflammatory cell infiltration did not precede the formation of the lung lesions but was rather accompanied with late tumor development. These data support a model where the continuous regenerative process initiated by oncogenic B-RAF-driven alveolar disruption provides a tumor-promoting environment associated with chronic inflammation.

## Introduction

The Ras-mitogen-activated protein kinase (MAPK) pathway is a key signaling pathway that is involved in the regulation of normal cell proliferation, survival, growth, differentiation, and apoptosis [1] [2] [3]. Activating mutations and deregulated expression of the components of this signaling network are the hallmarks of several human cancers and other human diseases [1]. To activate the MAPK signaling cascade, active Ras recruits RAF serine/threonine kinases to the plasma membrane where they become activated by several mechanisms [3]. Active RAF then phosphorylates MEK (for MAPK and extracellular signal-regulated kinase [ERK] kinase), which subsequently phosphorylates ERK to relay extracellular stimuli to the nucleus. There are three RAF-family members, A-, B- and C-RAF. Among these, B-RAF is the most frequently mutated RAF oncogene in human cancer [4]. Activating B-RAF mutations were found in melanoma, colorectal, papillary thyroid, ovarian and non-small-cell lung cancers (NSCLC) [5] [6]. A valine-glutamate substitution at residue 600 is the most prevalent type of B-RAF mutation (B-RAF V600E). This mutant exhibits a hyperactive kinetic function compared to the wild type form and accounts for ∼90% of all B-RAF mutations [7].

Deregulation of the mitogenic cascade is found in 50% of lung cancer patients [8]. Several studies using transgenic mouse models to understand the link between perturbations of MAPK signaling and lung cancer were made [9]. These models faithfully mimicked human NSCLC pathogenesis and predicted alveolar epithelial type II or Clara cells as the potential cells of origin for neoplastic transformation [9]. Based on the occurrence of B-RAF V600E mutations in NSCLC patients, we and others have recently begun to evaluate the role of this type of B-RAF mutation (B-RAF V600E) in lung tumor initiation and progression using mouse models. One of these models employed a knock-in strategy in which the oncogenic B-RAF allele is activated by infection of lungs with adenovirus expressing Cre-recombinase [10]. These mice developed benign neoplastic adenomas in the lung that show some signs of senescence in the course of disease progression. However, in this study the tumor-initiating cell could not be identified due to the promiscuous target cell specificity of the activating virus [10]. In a second study Ji *et al.* used an inducible rat specific CCSP promoter that targets both bronchiolar Clara cells as well as a fraction of type II cells [11] for expression of B-RAF V600E. However, lung tumor formation in this model was only achieved in an *Ink4A/Arf*-/- background [12].

Here we have instead employed the Surfactant Protein C (SpC) promoter to drive B-RAF V600E expression in type II pneumocytes. We have previously shown that these cells respond to C-RAF mediated activation of the mitogenic cascade with benign adenoma formation in the absence of additional genetic lesions [12], [13]. In contrast to these earlier works, expression of B-RAF V600E in type II pneumocytes did not readily elicit lung tumor formation but predominantly resulted in airspace enlargement accompanied with tissue remodeling and chronic inflammation. Tumor formation in our transgenic mice was a late event and displayed low penetrance.

## Materials and Methods

### Ethics Statement

All animal experiments were performed in accordance with German legislation on protection of animals and were approved by the local governmental animal care committee (Regierungspraesidium Darmstadt) (permit number V54-19c20/15-B2/233).

### Generation of transgenic mice

Mice were housed in air-filtered units with a 12-hour dark/light circle and with the access to food and water *ab libitum*. SpC-B-RAF V600E plasmid was generated by releasing the 2.5 Kb human B-RAF V600E cDNA from pKS-h B-RAF V600E (unpublished) by digestion with *NotI* restriction enzyme and inserted into SPC/SV40 plasmid (kindly obtained from Jeffrey Whitsett) that was previously digested with *Bam HI* endonuclease. Prior to ligation, both vector and insert were blunt-ended with T4 DNA polymerase. Correct orientation of the insert was tested by digestion with *XhoI* and *SalI* endonucleases and gel electrophoresis analysis. SpC-B-RAF V600E expression cassette (6.2 Kb) was resolved in a low melting agarose gel after digestion with *Hind III* restriction enzyme. The purified fragment was then injected into the pronucleus of fertilized eggs of FVB/n mice. Three positive founders that showed germ line transmission were obtained. All founders were backcrossed to C57Bl/6 for more than six generations before the onset of experiments.

### Genotyping

Genotyping of transgenic mice was performed via PCR using tail lysate as DNA template. To detect the SpC-B-RAF V600E transgene, SPC-BRAF S1 (5′GGA GGA GGT GTG GAA TAT CAA AC-3′) and SPC-BRAF AS1 (5′CCA ACA CTT CCA CAT GCA ATT C-3′) primers were used. The PCR conditions applied were the following: 95°C denaturation for 5 minutes followed by 35 cycles with 95°C denaturation for 30 seconds, 60°C annealing for 30 seconds and 72°C elongation for 1 minute with a unique final step at 72°C for 10 minutes. The final PCR product (610 bp) was resolved in a 1% agarose gel.

### Alveolar Type II cells isolation

Alveolar Type II cells were isolated from wild type and SpC-B-RAF V600E transgenic mice as previously described [14]. Briefly, lungs were lavaged with 10 ml PBS and tissues were digested with collagenase/dispase and subsequently minced. The cell suspension was filtered through 100-, 40- and 10 µm cell strainers and centrifuged at 130 g for 8 minutes. The cell pellet was resuspended in DMEM supplemented with 10% FCS and negative selection for macrophages/leucocytes was carried out by incubation on CD45- and CD16/32-precoated Petri dishes for 2 hours at 37°C. The cell suspension was centrifuged at 130 g for 8 minutes and the cell pellet was snap frozen for further studies.

### Reagents and antibodies

For immunohistochemistry, primary antibodies against the following proteins were used: Aquaporin 5 (AQP-005, Alomone Labs, 1∶1000); BRAF (H-145, Santa Cruz, 1∶250); CCSP (sc-9772, Santa Cruz, 1∶2500); CD3 (clone SP7, Lab Vision; 1∶1000); CD45 (BD Pharmingen, 1∶20); Active Caspase-3 (Asp175) (#9664, Cell Signaling, 1∶200); F4/80 (ab6640, Abcam, 1∶100); Ki67 (VPK452, MM1 Vector laboratories, 1∶50); pan-Cytokeratin (Z0622, Dako, 1∶500); Pax5 (BD Transduction Laboratories, 1∶1000); phospho-ERK (#4376, Cell Signaling, 1∶100); phospho-STAT3 (D3A7, Cell Signaling, 1∶100); Pro SP-C (gift of Jeffrey A. Whitsett); Raf1 (E10, Santa Cruz, 1∶100); TTF-1 (M3575, Dako; 1∶100); Vimentin (C-20, Santa Cruz; 1∶200). For Western blot analysis antibodies against the following proteins were used: Active ß-catenin (8E7, Millipore, 1∶2000); BRAF (H-145, Santa Cruz, 1∶1000); E-cadherin (#3195, Cell Signaling, 1∶1000); ERK 1/2 (#4695, Cell Signaling, 1∶1000); p120^ctn^ (6H11, Santa Cruz, 1∶1000), phospho-ERK (#4376, Cell Signaling, 1∶1000); phospho-STAT3 (D3A7, Cell Signaling, 1∶2000); Pro-SPC (gift of Jeffrey A. Whitsett); total STAT3 (124H6, Cell Signaling, 1∶1000); ß-catenin (BD Pharmingen, 1∶1000); Vimentin (R28, Cell Signaling; 1∶1000); ß actin (I10, Santa Cruz, 1∶3000).

### RNA isolation and RT-PCR analysis

RNA was isolated from total lung of 16.5 days-old embryos and adult mice of different ages or from isolated lung type II cells using Trizol solution (Invitrogen). Isolated type II cells from different animals were pooled prior to RNA isolation. RNA was treated with RNase-free-DNase I and cDNA synthesis was performed using the First-Strand cDNA Synthesis Kit (Fermentas) according to the manufacturer's instructions. Control samples were run without reverse transcriptase in the reaction. To detect SpC-B-RAF V600E transgene expression, HS-BRAF-S (5′-GTC ATC TTC ATC CTC AGA AG-3′) and HM-BRAF-AS (5′-TTC AAC ATT TTC ACT GCC AC-3′) primers were used. For the detection of endogenous murine B-RAF transcript, MM-BRAF-S (5′-CAT CTT CTT CCT CAT CCT CG-3′) and HM-BRAF-AS primers were used. PCR conditions applied were the following: initial denaturation at 94°C for 5 minutes, followed by a cycle repeated different number of times with denaturation at 94°C for 30 seconds, annealing at 61°C for 30 seconds and elongation at 72°C for 1 minute followed by a final step at 72° for 5 minutes. Real time PCR was performed using the DyNAmo™ HS SYBR® Green qPCR Kit (Finnzymes) in a Roto- Gene 2000 detection system (Corbett Research). ß actin or Hypoxanthine-Guanine Phosphoribosyltransferase (HPRT) were used as standard controls. Primer sequences are reported in Table S1.

### Western blot analysis

Lung tissues were lysed in an ice-cold RIPA buffer (50 mM Tris-HCl, pH 8.0, 150 mM NaCl, 0.1% SDS, 0.5% deoxycholate [DOC], 1% Nonidet P-40 with protease and phosphatase inhibitor cocktails) using an Ultra-Turrax homogenizer in a cold environment. Isolated lung type II cells were lysed in an ice-cold RIPA buffer by incubating on ice for 20 minutes. After centrifugation at 11,000 rpm for 10 minutes, supernatants were transferred into new tubes and protein concentration was measured using the BCA protein assay kit (Thermo Scientific). The remaining protein extracts were mixed with 4× laemmli buffer, heated at 95°C and vortexed for 10 seconds. 30 µg proteins were resolved by SDS-PAGE and transferred to nitrocellulose membrane. After 1 h blocking with 5% non-fat-dry-milk in NET-gelatine (50 mM Tris/HCl pH 7.4, 5 mM EDTA, 0.05% Triton X-100, 150 mM NaCl, 0,25% gelatine) blots were incubated overnight at 4°C with primary antibodies, washed three times with NET-gelatine and incubated for 1 h at room temperature with peroxidase coupled secondary antibodies (GE Healthcare). After three washes with NET-gelatine, blots were subsequently developed using an ECL kit (Thermo Scientific).

### Histopathology and immunostainings

Animals were sacrificed and lungs were fixed under 25 cm water pressure with 4% buffered paraformaldehyde and paraffin embedded. 6 µm-cut sections were deparaffinized, rehydrated in graded series of alcohol and haematoxylin-eosin stained. For analysis of airspace enlargements lungs from wild type and transgenic mice were evaluated blindly by two independent examiners. Tissue samples were graded in the following manner: 1) Micro- and macro- scopically normal; 2) Macroscopically normal, but microscopically abnormal [airspace enlargement]; 3) Macroscopic abnormalities [bulb formation]. For immunostainings, sections were heated in 10 mM sodium citrate buffer (pH 6) for 10–20 minutes using a microwave oven. Endogenous peroxidase activity was blocked by incubation with 1–3% H_2_O_2_ in methanol or PBS. Unspecific binding of the antibodies was prevented by incubation with 3–5% serum and 0.1% Triton-X in PBS for 1 hour at room temperature. All primary antibodies were incubated overnight at 4°C. For phospho-ERK staining, Tris-buffer-saline containing 0.1% Tween 20 (TBST) was used instead of PBS. For immune detection of CD45 and F4/80 proteins, antigen retrieval was done by incubating the lung sections with 20 µg/ml proteinase K in TE buffer (pH 8) for 20 and 7 minutes, respectively. Biotinylated secondary antibodies (DakoCytomation) were incubated for 90 minutes at a dilution of 1∶200 in blocking solution. ABC reagent was applied (Vectastain Elite ABX Kit, Vector Labs) and developed in diaminobenzidine (DAB). Sections were counterstained with hematoxylin and dehydrated before mounting. For Masson's Trichrome staining, the Accustain Trichrome Stain kit (Sigma Aldrich) was used according to the manufacturer's instructions. For Alcian Blue staining, lung sections were deparaffinized, rehydrated in graded alcohols and incubated in Alcian Blue (pH 2.5) solution for 30 minutes at room temperature. After washing in tap water for 5 minutes, sections were counterstained with Nuclear Fast Red for 5 minutes, washed with tap water for 3 minutes, dehydrated and mounted with entellan.

### Analysis of mutations

Screening of mutations in tumor samples was carried out as previously described [15]. The following genes were analyzed for mutations: *EGFR* exon 19, 20 21; *K-RAS* exon 1 and 2; *p19^ARF^* exon 1; *p16^INK4a^* exon 1; *p16^INK4a^* exon 2; *p16^INK4a^* exon 3; *p53* exon 5, 7 and 8; *LKB1* exon 1, 2 and 6. Primers that were used are listed in Table S2.

### Statistical analysis

All statistical analyses were performed using GraphPad Prism4 software. Log-rank analysis was used to compare the survival rate of different group of mice. Student's *t*-test (two-tailed) was used to compare two groups (*P*<0.05 was considered significant).

## Results

### Expression of B-RAF V600E in alveolar type II cells leads to airspace enlargements in mice

**Figure 1:**
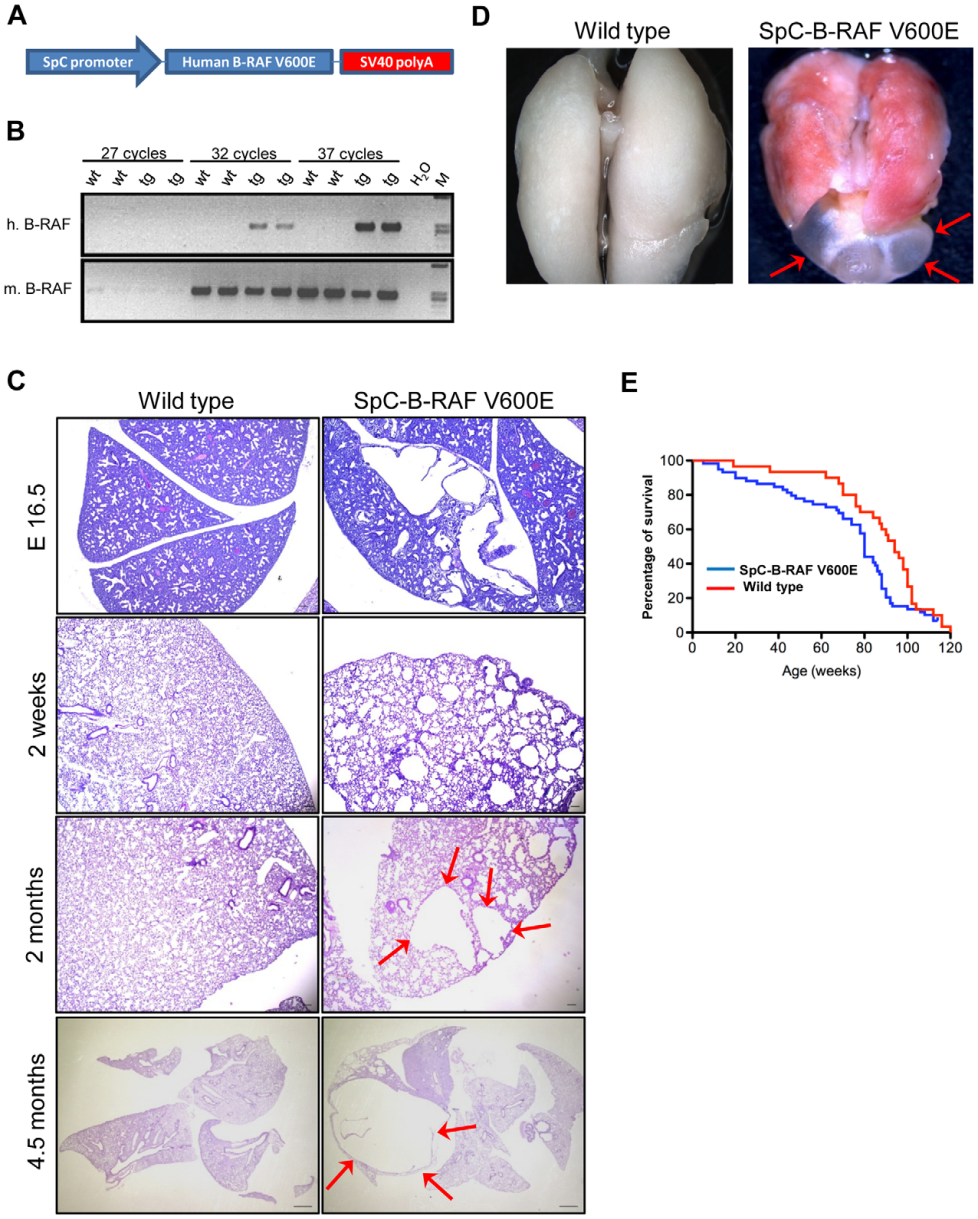
Induction of alveolar enlargements in mice with targeted expression of mutant B-RAF (V600E) in lung alveolar type II cells. (A) Schematic representation of the targeting vector. (B) Semi-quantitative RT-PCR shows transgenic- and endogenous- B-RAF expression in lungs of two wild type and transgenic embryos (E16.5), (cycles = PCR cycle; M = marker; h = human; m = mouse). (C) Paraffin embedded hematoxylin and eosin (H&E) stained lung sections from mice of different ages show airspace enlargements (arrows) in transgenic animals, (scale bar = 100 µm for the first, second and third panel; 1 mm for the forth panel). (D) Whole lung photographs of 4.5 months old wild type and transgenic mice show a macroscopic bleb (arrows). (E) Kaplan-Meier survival curve, (log-rank analysis *P*<0.05; SpC-B-RAF V600E *n* = 59; wild type *n* = 30).

**Figure 2:**
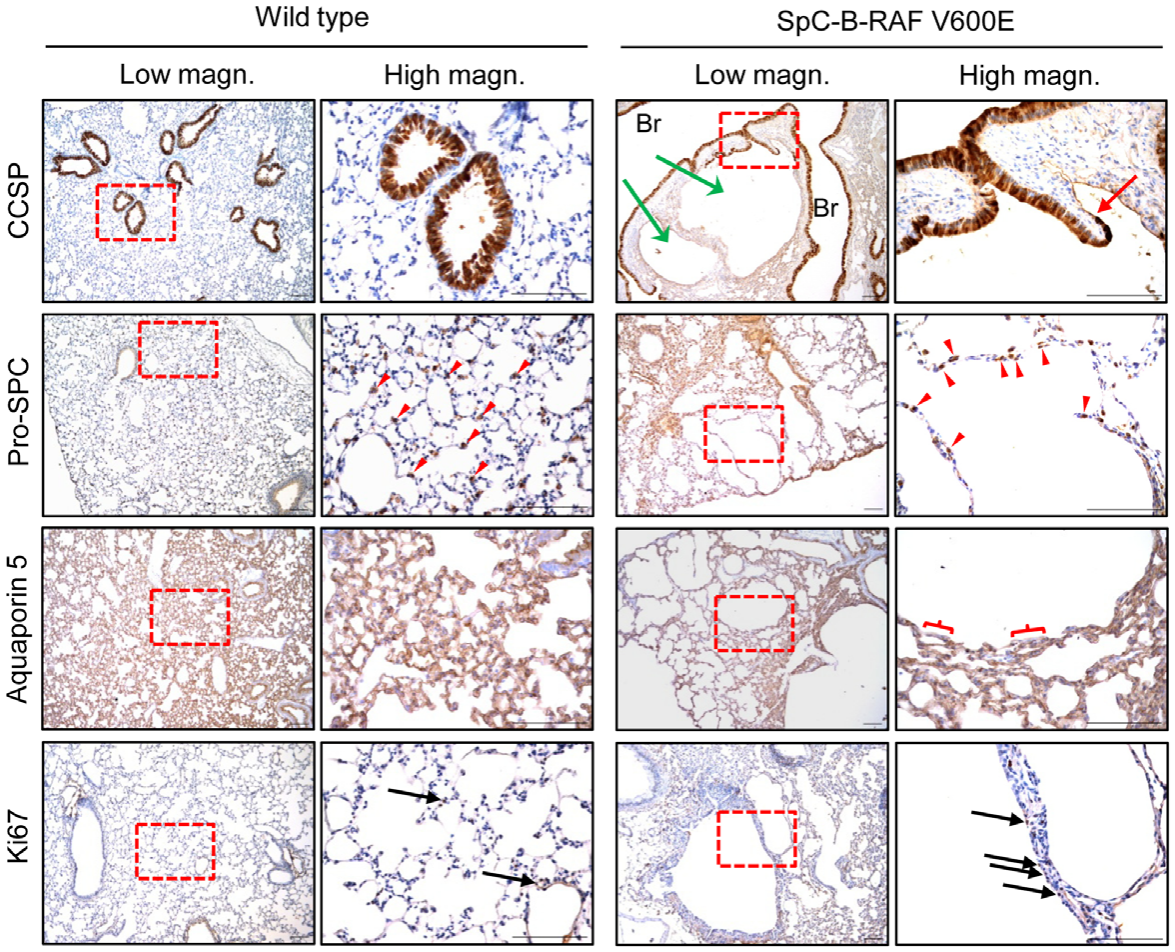
Immunohistochemical analysis of lung lesions for lung differentiation markers. Paraffin embedded lung sections from wild type and transgenic mice at 2 months of age were stained for indicated markers; (green arrows point out airspace enlargements), (the red arrow marks the bronchio-alveolar junction), (red-parenthesis indicates a AQP5 negative sector), (red arrowheads point to Pro-SPC expressing cells), (black arrows highlight Ki67 positive cells), (Br = bronchiole), (hematoxylin was used as a counterstain), (right panel pictures are high magnification of the red insets); scale bar = 100 µm.

**Figure 3:**
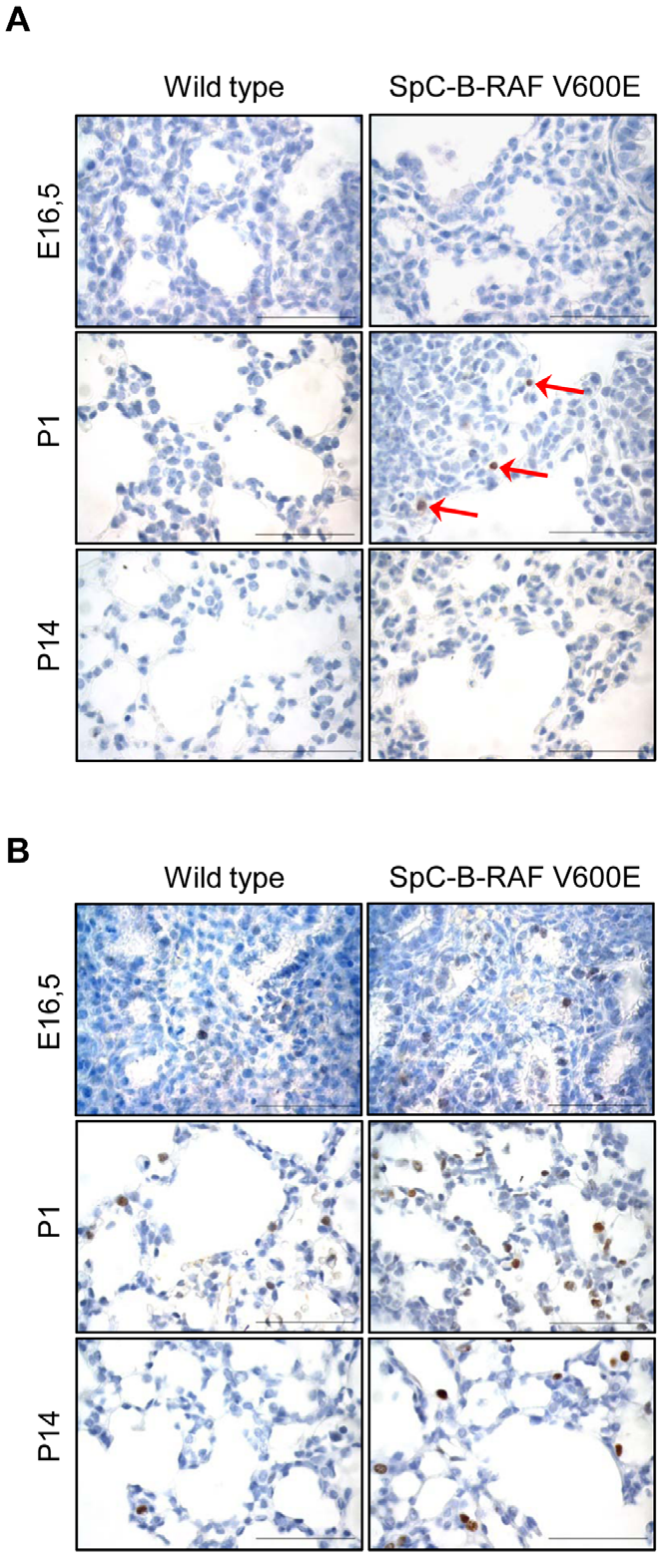
Analysis of cell death and cell proliferation in transgenic mice. (A) Active caspase 3 staining of control and transgenic animals, (arrows indicate positive cells). (B) Ki67 staining (brown) of control and transgenic animals. (E = embryonic, P = post-natal), (hematoxylin was used as a counterstain); scale bar = 50 µm.

We have generated three transgenic mouse founders for the expression of B-RAF V600E under the control of the human SpC promoter (Figure 1A). SpC-B-RAF V600E transgenic mice were born in the expected mendelian ratios, were fertile and did not show any gross morphological or behavioral abnormalities. Analysis of transgene mRNA expression levels among the founders showed comparable results (Figure S1A). Histopathological analysis of lung sections from adult mice revealed airspace enlargements with various degrees in all the founders compared to control animals (Figure S1B). Interestingly lung tumor formation was not detected in any of these founders (Figure S1B). Based on the highest penetrance of the airspace enlargements, we have selected the founder line number 3 for further studies. Consistent with the early expression of the transgene (Figure 1B), we found airspace enlargements in the embryonic lung sections (Figure 1C) that persisted in both post-natal and adult lungs of the transgenic animals (Figure 1C). Some of these alveolar disruptions increased in size over time forming macroscopic air-filled structures reminiscent of human bullous-emphysema (Figure 1C–1D and S2A–S2B). The presence of these lesions affected the life span of animals (Figure 1E). Consistent with the diminished levels of transgene expression in adult mice (Figure S3A), neither total B-RAF nor phospho-ERK protein levels altered between the wild type and transgenic mice (Figure S3B). Immunohistochemical analysis of cells lining these macroscopic lesions showed presence of epithelial cell types from the distal lung, including Clara cells, type I and type II pneumocytes (Figure 2). Notably, these lesions were often found to be continuous with bronchio-alveolar junctions (Figure 2). Programmed cell death and tissue regeneration play an important role during lung morphogenesis. We therefore screened lung sections from SpC-B-RAF V600E transgenic mice of different ages for apoptosis and proliferation markers. We detected active caspase 3 staining in lung sections from transgenic mice only at postnatal day 1 (Figure 3A). There was no change in cell proliferation assessed by Ki67 immunostaining at this age (Figure 3B). However, we found increased cell proliferation in lungs of transgenic mice at postnatal day 14 (Figure 3B). Taken together these data suggest that embryonic expression of B-RAF V600E in alveolar type II cells impairs lung development leading to airspace enlargements.

### Occurrence of tissue remodeling in the lung of SpC-B-RAF V600E transgenic mice

**Figure 4:**
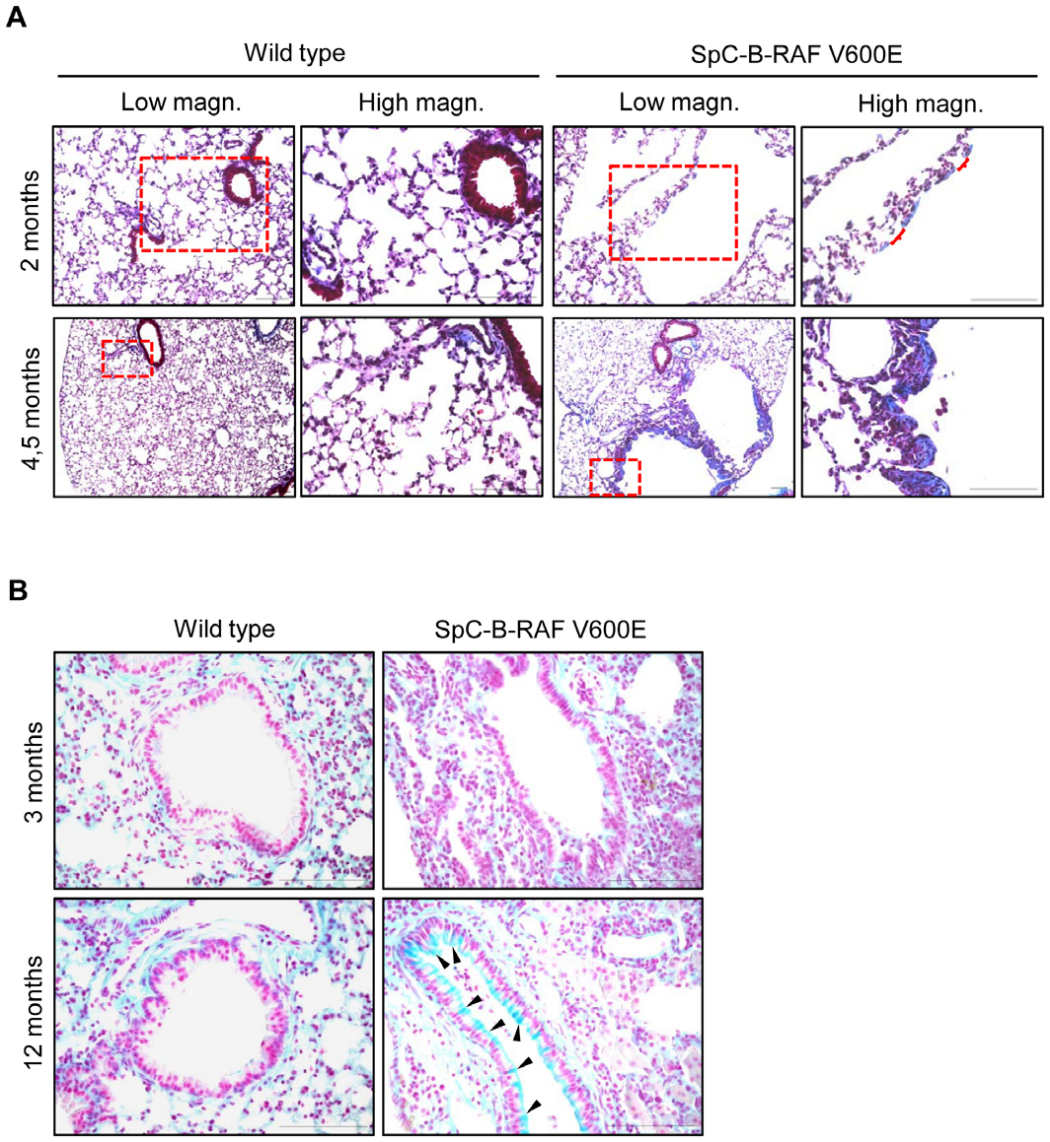
Collagen accumulation and Goblet cells hyperplasia in aged SpC-B-RAF V600E transgenic mice. (A) Masson's Trichrome staining shows collagen (blue), red-parenthesis indicates Masson's Trichrome negative parts, (the right panel pictures are high magnification of the red insets). (B) Representative pictures of Alcian blue stained paraffin embedded lung sections from wild type and transgenic mice at 3 and 12 months of age, respectively, black arrowheads denote Alcian Blue-positive (blue) regions indicative of increased mucus production; scale bar = 100 µm.

To further characterize the B-RAF V600E induced lung lesions, we examined lung sections from wild type and transgenic animals for the presence of collagen accumulation and mucus secretion that are the prominent features of chronic lung diseases [16]. Interestingly we detected progressive deposition of collagen (Figure 4A) in non-epithelial lining sectors of these lesions (Figure 2, red parenthesis) indicating an ongoing tissue remodeling. In addition to collagen accumulation we also found an age-related increase in mucus secretion in transgenic mice (Figure 4B).

**Figure 5:**
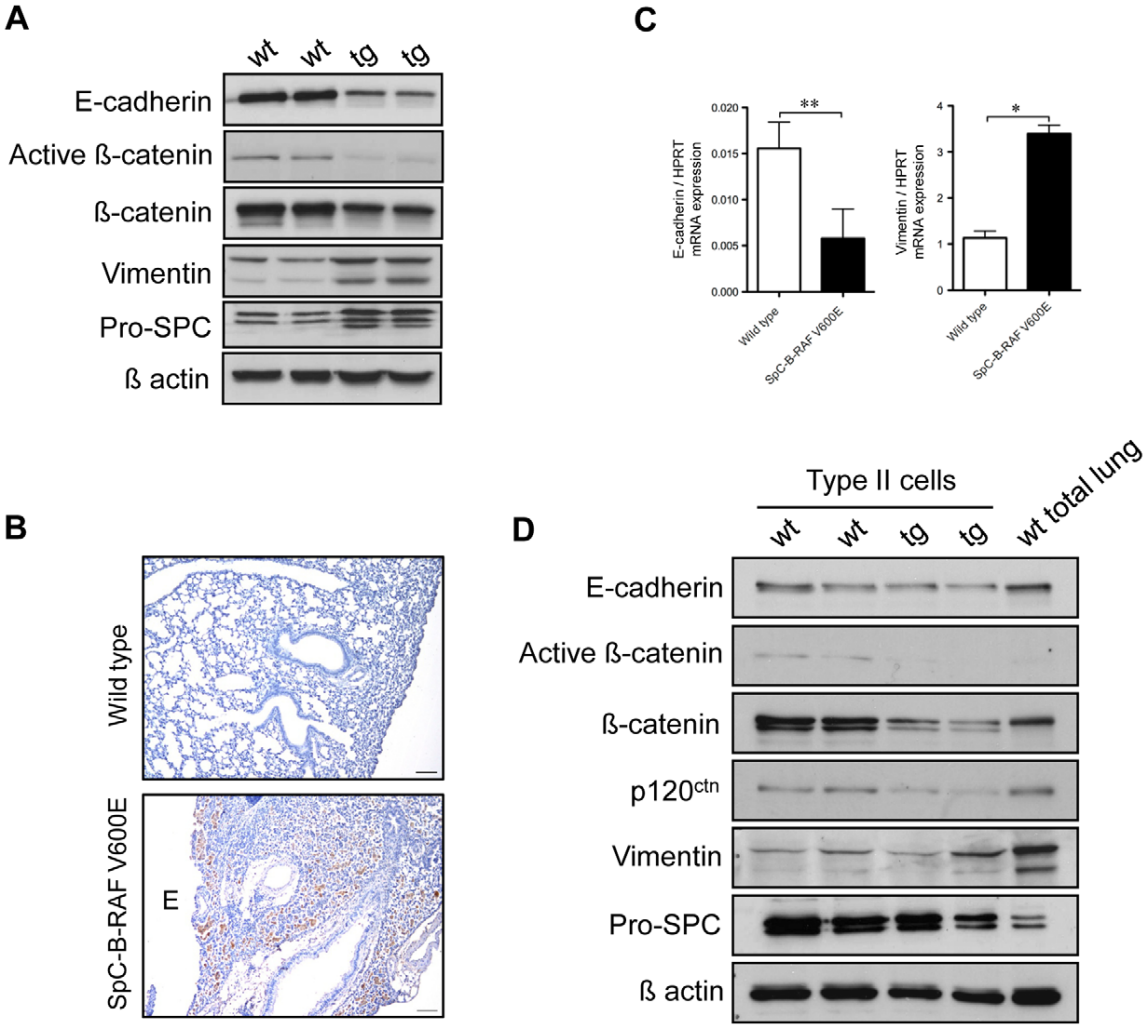
Analysis of Epithelial-Mesenchymal-Transition (EMT) in SpC-B-RAF V600E transgenic mice. (A) Total lung protein lysates from two wild type (wt) and transgenic (tg) littermates (17 and 21 months old) were gel separated and immunoblotted with the indicated antibodies. (B) Immunostaining of lung sections from wild type and SpC-B-RAF V600E transgenic mice for Vimentin (brown), (E = airspace enlargement), hematoxylin was used as a counterstain, scale bar = 100 µm. (C) Analysis of E-cadherin and Vimentin mRNA levels in alveolar type II cells isolated from wild type and SpC-B-RAF V600E transgenic mice (5 months old) by Real time PCR, data represent mean+SEM, (*t*-test * = *P*<0,05, ** = *P*<0,01, *n* = 3–4). (D) Protein lysates of isolated type II cells from two wild type (wt) and transgenic (tg) littermates (20 months old) were gel separated and immunoblotted with the indicated antibodies.

Epithelial-Mesenchymal-Transition (EMT) was often shown to be associated with chronic human lung diseases such as COPD and pulmonary fibrosis [17], [18], [19], [20]. We therefore analyzed total lung tissue lysates of SpC-B-RAF V600E transgenic mice, which harbored macroscopic lesions (grade 3) (Figure S2A–S2B) with known EMT markers. We found reduced expression of E-cadherin, ß-catenin and increased expression of Vimentin, indicating EMT induction (Figure 5A), although we cannot rule out the induction of an alveolar maintenance program in alveolar type II cells [21]. Expression of Vimentin was not solely localized next to the lesions but also abundantly found in fibrotic lung parenchyma (Figure 5B). Since the transgenic lungs show airspace enlargements and inflammation, these results could simply reflect the relative changes in cell populations in the lung rather than an EMT phenomenon. We therefore analyzed the same markers in isolated alveolar type II cells from mice that in this case did not show macroscopic lesions by Real-Time PCR and immunoblotting. We found significantly reduced E-cadherin and increased Vimentin mRNA levels in isolated type II cells from SpC-B-RAF V600E mice (Figure 5C). Different from the mRNA data, we detected equal to mildly decreased levels of E-cadherin in protein lysates from transgenic animals (Figure 5D). In contrast, variable levels of Vimentin expression were detected between the samples (Figure 5D). As in the case of the total lung lysates we observed strongly reduced levels of ß-catenin in type II cells from mutant mice (Figure 5A and 5D). In addition to ß-catenin, the levels of p120^ctn^ were also reduced (Figure 5D). ß-catenin is a key signaling mediator of the canonical WNT signaling [22]. Interestingly, in both total lung and isolated type II cells protein lysates from SpC-B-RAF V600E mice we detected diminished levels of active ß-catenin expression suggesting an inhibition of the canonical WNT signaling pathway (Figure 5A and 5D).

### Induction of inflammation in SpC-B-RAF V600E transgenic mice

**Figure 6:**
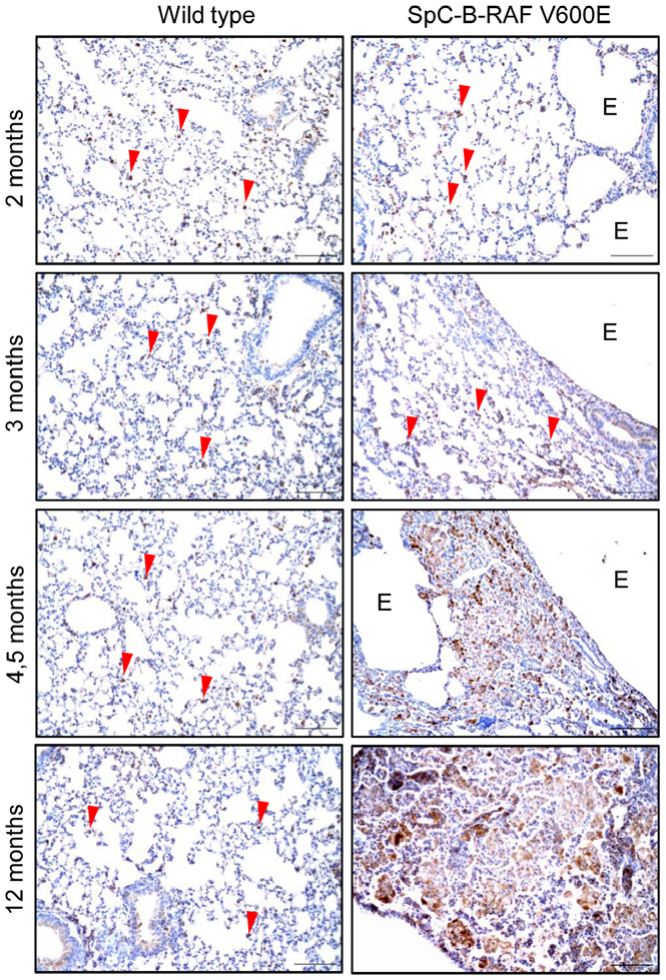
Progressive accumulation of macrophages in the lung of SpC-B-RAF V600E transgenic mice. Paraffin embedded lung sections from mice with the indicated genotypes and ages stained for F4/80 (brown), E = airspace enlargement, arrowheads show diffused alveolar macrophages, hematoxylin was used as a counterstain, scale bar = 100 µm.

**Figure 7:**
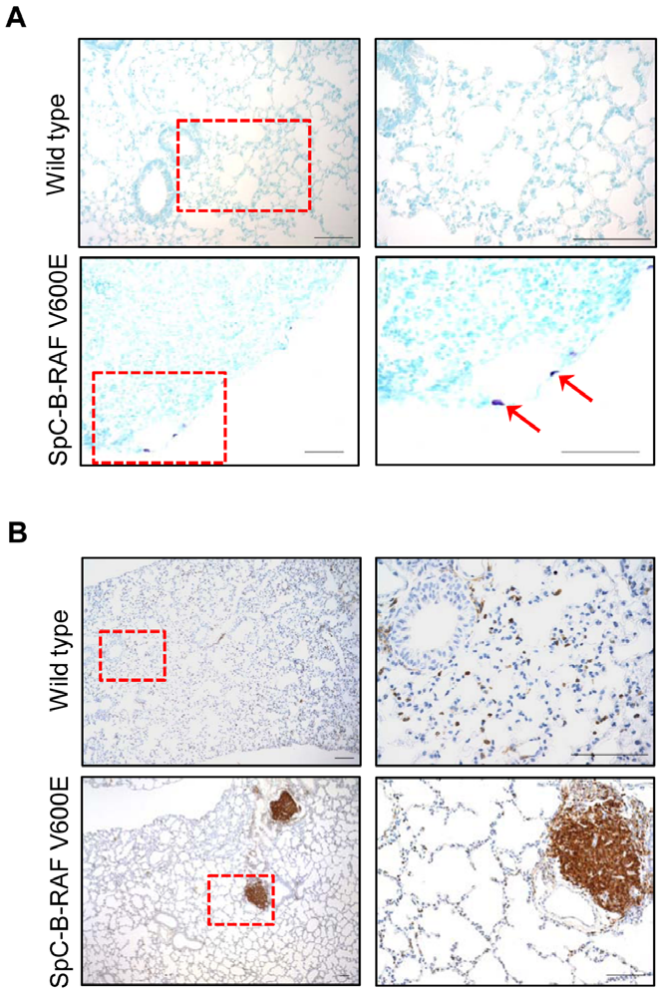
Infiltration of immune cells in B-RAF V600E-induced lung lesions. (A) Toluidine blue staining identifies mast cells (purple) in the epithelial lining of a lesion. (B) Staining of paraffin embedded lung sections from wild type and transgenic mice for CD45 (brown), hematoxylin was used as counterstain, the right panel pictures are high magnification of the red insets; scale bar = 100 µm.

**Figure 8:**
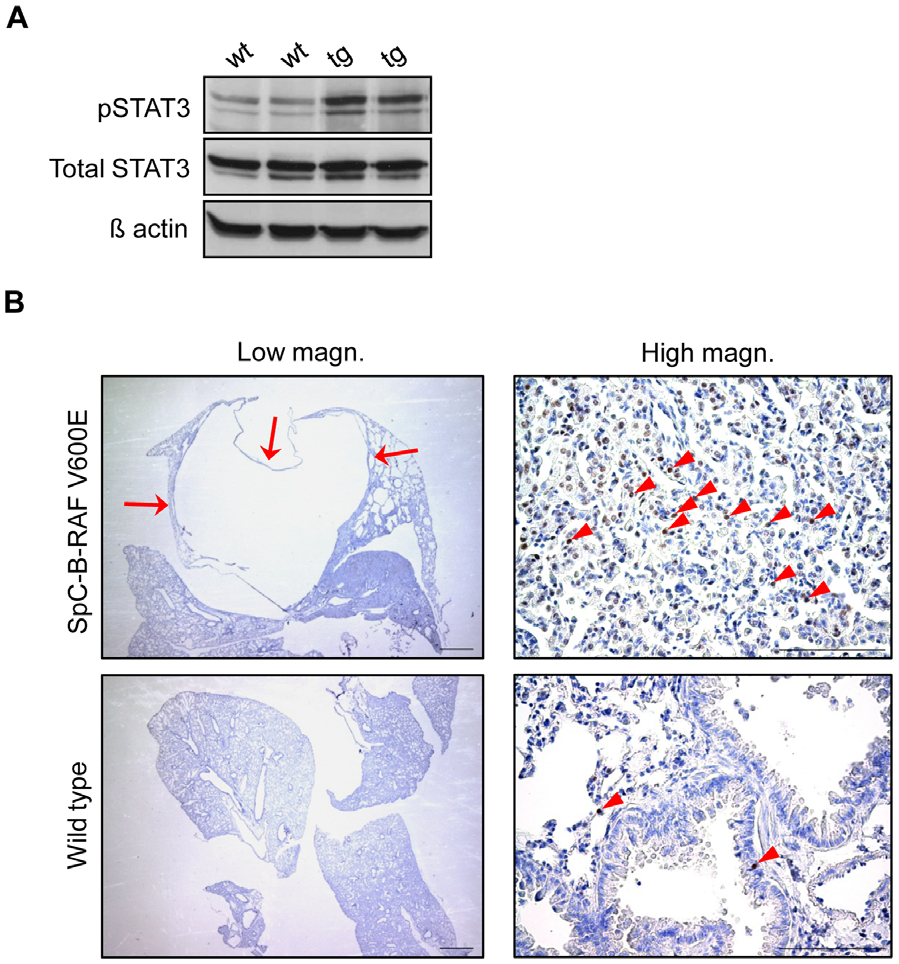
Activation of STAT3 signaling in SpC-B-RAF V600E transgenic mice. (A) Total lung protein lysates from two wild type (wt) and transgenic (tg) littermates (17 and 21 months old) were gel separated and immunoblotted with the indicated antibodies. (B) Phospho-STAT3 immunostaining of lung sections shows accumulation of STAT3 positive cells in transgenic mice (arrows indicate a macroscopic bleb, arrowheads point to Phospho-STAT3 positive cells), the right panel pictures are high magnification of the red insets, hematoxylin was used as a counterstain, scale bar = 1 mm in the right and 100 µm in the left panel pictures.

Chronic inflammation is a prominent feature of degenerative lung diseases in human [23]. To assess the involvement of immune cells in lungs of SpC-B-RAF V600E transgenic mice we screened lung sections from young (1–3 months) and aged (4–28 months) mice using different immune cell markers. Progressive accumulation of infiltrating macrophages was observed in the vicinity of the lesions (Figure 6). In some cases, this massive accumulation led to granulomas formation in transgenic mice (Figure 6). Interestingly mast cells were detected in the walls lining the enlarged air spaces (Figure 7A). Moreover, we found lymphoid follicles consisting of T- and B-cells in lungs of SpC-B-RAF V600E transgenic mice (Figure 7B and Figure S4). In extreme cases, these lymphoid aggregates reached very large sizes occupying most of the air exchange spaces of the entire lung (Figure S5A). These CD45 positive clusters were surrounded by intense collagen accumulations (Figure S5B). In order to evaluate the potential role of the adaptive immunity in the development of the B-RAF V600E-induced lung lesions, SpC-B-RAF V600E single transgenic mice were crossed with *RAG1*-null mice that lack mature T- and B- lymphocytes [24]. Histological examination of lung sections from compound mice (SpC-B-RAF V600E/*RAG1* KO) demonstrated the persistence of these lesions indicating that T- and B- cells are not involved in the lung pathogenesis in our system (Figure S6A and S6B). In order to identify the mechanisms by which B-RAF V600E triggers inflammation, IL-6/signal transducer and activator of transcription 3 (STAT3), a well known inducer of inflammation [25], was studied. Western blot analysis of lung protein lysates showed up-regulation of phospho-STAT3 in transgenic animals (Figure 8A). Immunostaining of lung sections with phospho-STAT3 specific antibody showed similar results indicating the activation of STAT3 signaling in SpC-B-RAF V600E mice (Figure 8B).

### Delayed tumor formation in SpC-B-RAF V600E transgenic mice

**Figure 9:**
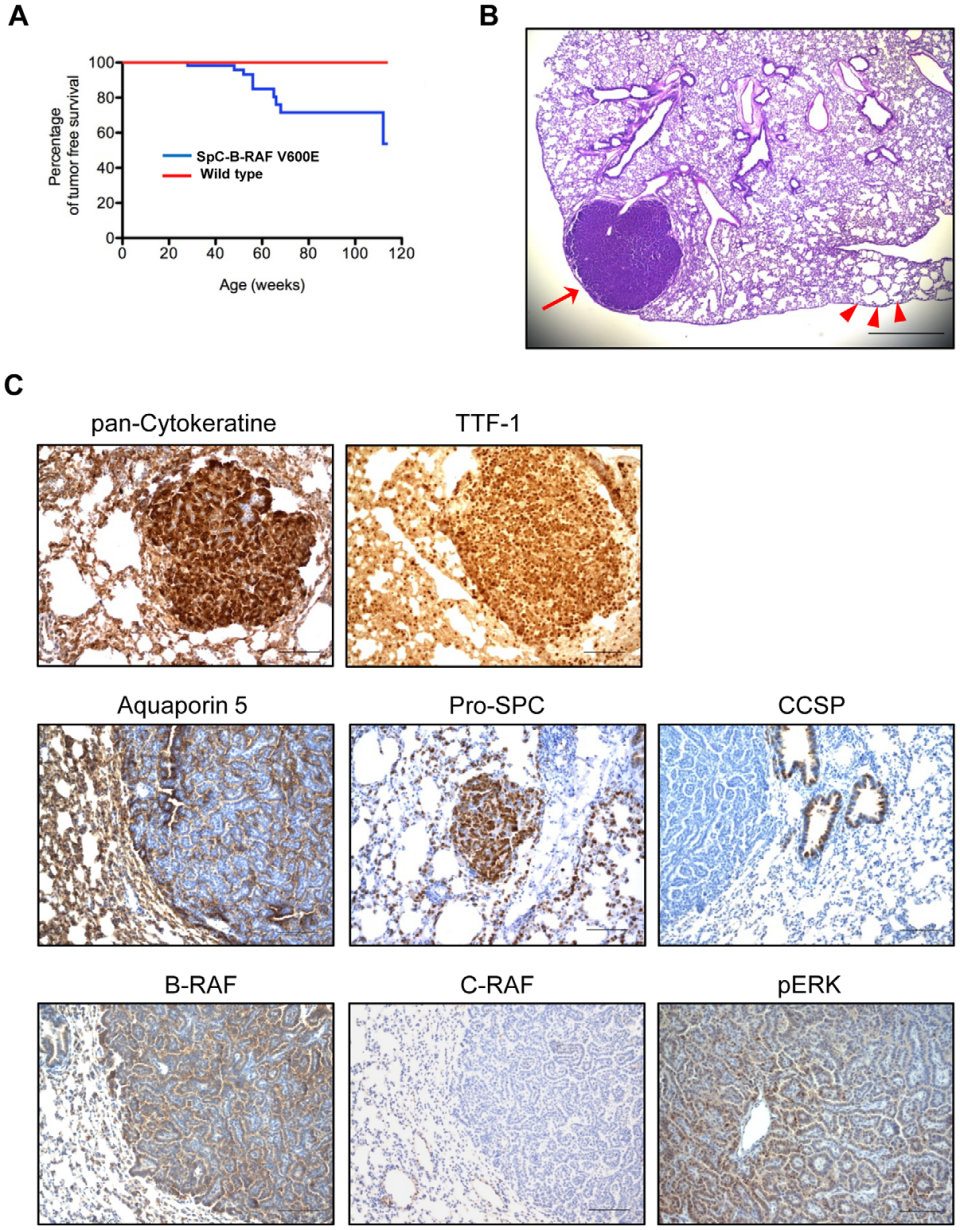
Lung tumor formation in SpC-B-RAF V600E transgenic mice. (A) Tumor incidence as a function of age, log-rank analysis for wild type (*n* = 59) and SpC-B-RAF V600E (*n* = 102) transgenic mice, *P*<0.05. (B) H&E staining of lung section from a one year old SpC-B-RAF V600E transgenic mouse shows coexistence of the lung lesions (arrowheads) with a lung tumor (arrow); scale bar = 500 µm. (C) Tumor bearing lung sections were stained with the indicated markers, hematoxylin was used as counterstain; scale bar = 100 µm.

As there is a large body of clinical evidence that human chronic lung diseases may be linked with the late onset lung cancer [26] [27], we looked at a cohort of aged mice for lung tumor formation. In comparison to wild type control littermates, we detected single focal lung tumors with low incidence (10%) in SpC-B-RAF V600E transgenic mice (Figure 9A and 9B). Notably, tumors were always found in lungs with airspace enlargements (Figure 9B). Lung tumors stained positive for TTF-1, Pro-SPC, and Aquaporin-5 and negative for CCSP indicating a type II pneumocyte origin (Figure 9C). Immunohistochemical analysis of tumors with the components of mitogenic cascade signaling showed positive staining for both B-RAF and phospho-ERK1/2 but not for C-RAF (Figure 9C). Low incidence and late latency of lung tumors from SpC-B-RAF V600E mice are in the favor of a spontaneous origin. We therefore screened tumor samples for somatic mutations that are commonly found in human and mouse NSCLC [4]. Interestingly the majority of the tumors were negative for these mutations (Figure S7).

## Discussion

Mutations in the components of the mitogenic cascade play an important role in both developmental syndromes and neoplastic diseases [28]. Individuals with these syndromes often have mutations in this signaling pathway that predisposes them to cancer too [28]. NSCLC is the leading cause of cancer related death worldwide and frequently harbors activating mutations in the MAPK pathway [8]. A fraction (∼2%) of NSCLC patients harbors B-RAF mutations [4] and several studies earlier attempted to model human NSCLC in mice. They have shown that conditional expression of B-RAF V600E rapidly induces lung adenoma formation [10], [12]. However, in our study, constitutive expression of this mutant B-RAF in type II pneumocytes did not elicit immediate tumor formation but predominantly caused airspace enlargements which were often associated with inflammation. Lack of tumor formation in SpC-B-RAF V600E transgenic mice in comparison to the SpC-C-RAF BxB mice [13] is intriguing, as both oncogenes are known to activate the mitogenic cascade. It is plausible that the diminished transgene expression caused by the induction of apoptosis during lung development precluded oncogenic transformation of alveolar type II cells. The absence of elevated phospho-ERK levels in transgenic mice supports such a hypothesis. Alternatively, as reported previously, induction of cellular senescence program by oncogenic B-RAF may restrict expansion of transformed cells [29]. However, we did not find detectable levels of p16^INK4a^, p21^WAF1^ and p19^ARF^, the common oncogene-induced senescence markers, protein expression neither by immunoblotting nor by immunohistochemistry in transgenic mice from different ages (data not shown).

In the present study, the expression of B-RAF V600E specifically in alveolar type II cells during lung development affected alveolar formation. Earlier studies showed that the canonical WNT signaling plays a crucial role during lung development [22]. Inhibition of WNT signaling by conditional knockout of ß-catenin in alveolar type II cells disrupted lung morphogenesis and caused respiratory failure due to dilated terminal structures in their lungs [22]. The airspace enlargements that we find in our transgenic mice may be related to the downregulation of WNT signaling that we detected in isolated type II pneumocytes. Interestingly, similar to oncogenic B-RAF, activation of oncogenic K-ras^G12D^, the most frequent mutation found in human NSCLC, was shown to impair lung morphogenesis by up-regulating Ras/MAPK antagonist Sprouty-2 in the absence of apoptosis [30]. It remains to be elucidated whether Sprouty-2 or other antagonists of the mitogenic cascade play a role in generation of developmental defects induced by B-RAF V600E in our transgenic model.

The airspace enlargements observed in embryonic lungs of SpC-B-RAF V600E transgenic mice were maintained in adult animals and were associated with tissue remodeling and chronic inflammation. Accumulations of collagen and mucus secretion that we found in transgenic lungs suggest that there is an ongoing tissue repair. However, persistence of the airspace enlargements throughout the lifetime of the animals indicates that there is an inefficient recovery of the lung epithelium. Inhibition of canonical WNT signaling as well as absence of full EMT in lungs of transgenic animals may account for incomplete repair. Chronic inflammation plays also an important role in tissue remodeling by promoting the release of cytokines by immune cells [31]. STAT3, a cytokine-induced gene related to pulmonary inflammation in human COPD, has been shown to be required for alveolar structure maintenance and function [32]. Persistent activation of the STAT3 signaling in murine respiratory epithelial cells was also shown to induce pulmonary inflammation and tumor formation in the lung [32]. Moreover STAT3 is a potent mediator of pro-inflammatory interleukin 6 (IL-6) that was shown to induce EMT phenotype in human breast cancer cells [33]. Abundant expression of activated STAT3 that we found in lungs of SpC-B-RAF V600E transgenic mice may indicate a role for STAT3 in the promotion of tissue remodeling and inflammation in our system.

Histopathological analysis of the infiltrating immune cells present in SpC-B-RAF V600E transgenic mice demonstrated the recruitment of inflammatory cells from both innate and adaptive immune system. The presence of lymphoid aggregates consisting of T- and B-cells raises the question whether there was a specific immune response to B-RAF V600E. We have previously described a T-cell specific epitope of B-RAF V600E [34] that could be involved in immune selection, suggesting that the immune response might be the driving force behind the development of these lesions in the transgenic animals. However, lack of immune cells in embryonic lungs with airspace enlargements and persistence of these lesions in lungs from SpC-B-RAF V600E/RAG1-null compound mice suggest that cells of the adaptive immune system are dispensable in the course of the disease. In fact, cells from the innate immune system; such as macrophages and mast cells were abundantly found in the lungs of transgenic animals. The progressive accumulation of macrophages as well as lymphoid aggregates might contribute to an elevated mucus production and increased damage observed in these lesions. In addition to macrophages, mast cells have important roles in innate immunity and tissue remodeling but were poorly studied in human COPD [35]. Our data suggest that mast cells might play a role in the progression of chronic lung degeneration and therefore should be examined in more detail in future.

It has been previously shown that the expression of B-RAF V600E in lung epithelial cells induced lung tumor formation. In contrast to these previous studies, we found that the early expression of mutant B-RAF in alveolar type II cells caused airspace enlargements in the absence of lung tumors. The differences between these models might result from a different targeting strategy, as well as different timing of oncogene induction [10] [12]. Nevertheless we detected lung tumors with low incidence only in aged transgenic animals. The presence of single focal tumors with low penetrance in transgenic mice suggests a spontaneous origin. However, the rate of spontaneous tumor formation in transgenic mice was significantly higher than in C57BL/6 mice [36]. Consistent with the link between inflammation and cancer, the increased number of spontaneous lung tumors found in SpC-B-RAF V600E mice might be due to inflammation that was often associated with the airspace enlargements.

## Supporting Information

Figure S1 **Comparable formation of airspace enlargements in different SpC-B-RAF V600E transgenic founder lines.** (A) Analysis of B-RAF V600E mRNA levels between the founders (2 weeks old) by Real-Time PCR, data represent mean+SEM, (*t*-test ns = not significant, *n* = 3). (B) Representative paraffin embedded H&E stained lung sections from wild type and transgenic mice (2 months old), right panel pictures are the high magnification of red insets, scale bar = 100 µm.
(TIF)

Figure S2 **Scoring of B-RAF V600E induced lung lesions in transgenic mice.** (A) Representative pictures of H&E stained lung sections from wild type and transgenic mice show airspace enlargements with different grades. The scoring was performed as follow: 1) Micro- and macro-scopically normal; 2) Macroscopically normal, but microscopically abnormal [airspace enlargement]; 3) Macroscopic abnormalities [bulb formation, pointed by arrows]. Scale bar = 1 mm for low magn. and 100 µm for high magn. pictures. (B) Incidence of the lung lesions with different grades, (*n* = 59 for wild type, *n* = 102 for SpC-B-RAF V600E).
(TIF)

Figure S3 **Diminished transgene expression and lack of phospho-ERK upregulation in SpC-B-RAF V600E mice.** (A) Semi quantitative RT-PCR analysis of total lung RNA samples from wild type (wt) and transgenic (tg) animals shows different levels of transgene expression between embryonic and adult mice, HPRT was used as an internal control. (B) Total lung protein lysates from two wild type (wt) and transgenic (tg) littermates were gel separated and immunoblotted; ages and antibodies are as indicated.
(TIF)

Figure S4 **Presence of T- and B- cells in CD45 positive clusters.** Lung sections from SpC-B-RAF V600E transgenic mice were stained for CD3 and PAX5 to identify T- and B- cells, respectively; hematoxylin was used as a counterstain; scale bar = 100 µm.
(TIF)

Figure S5 **Severe lung degeneration in an aged (17.5 months) mouse transgenic for B-RAF V600E.** (A) H&E staining of a lung section shows massive infiltration of leukocytes (red arrows). (B) Masson's Trichrome staining of the consecutive lung section reveals collagen-enriched regions (blue), the right panel are high magnifications of the yellow inserts; scale bar = 1 mm for the left and 100 µm for the right panel.
(TIF)

Figure S6 **Mature T- and B- cells are dispensable for the formation of SpC-B-RAF V600E-induced lung lesions.** (A) H&E staining of lung sections from wild type, RAG1 knock out (KO), SpC-B-RAF V600E single transgenic and SpC-B-RAF V600E/RAG1 KO compound animals show the persistence of airspace enlargements in SpC-B-RAF V600E single and compound mice, green arrows point to airspace enlargements. (B) Immunostaining of paraffin embedded lung sections from RAG1 KO single transgenic and SpC-B-RAF V600E/RAG1 KO compound animals for CD45 (brown), red arrows point to CD45 positive clusters, hematoxylin was used as a counterstain; scale bar = 100 µm.
(TIF)

Figure S7 **Lung tumors found in SpC-B-RAF V600E transgenic mice in general do not harbor mutations frequently present in human and mouse NSCLC.** Genomic DNA from lung tumors of SpC-B-RAF V600E transgenic mice were screened for the indicated genes, individual animal (ID) and Exon numbers are as indicated, wt = wild type, Hetero = heterozygous, Homo = homozygous, empty boxes represent untested samples.
(TIF)

Table S1 **Primers for Real time PCR.**
(DOCX)

Table S2 **Primers for the amplification of genomic DNA.**
(DOCX)

## References

[1] Roberts PJ, Der CJ (2007). Targeting the Raf-MEK-ERK mitogen-activated protein kinase cascade for the treatment of cancer.. Oncogene.

[2] Robinson MJ, Cobb MH (1997). Mitogen-activated protein kinase pathways.. Curr Opin Cell Biol.

[3] Wellbrock C, Karasarides M, Marais R (2004). The RAF proteins take centre stage.. Nat Rev Mol Cell Biol.

[4] Forbes SA, Bindal N, Bamford S, Cole C, Kok CY (2011). COSMIC: mining complete cancer genomes in the Catalogue of Somatic Mutations in Cancer.. Nucleic Acids Res.

[5] Davies H, Bignell GR, Cox C, Stephens P, Edkins S (2002). Mutations of the BRAF gene in human cancer.. Nature.

[6] Zebisch A, Troppmair J (2006). Back to the roots: the remarkable RAF oncogene story.. Cell Mol Life Sci.

[7] Wan PT, Garnett MJ, Roe SM, Lee S, Niculescu-Duvaz D (2004). Mechanism of activation of the RAF-ERK signaling pathway by oncogenic mutations of B-RAF.. Cell.

[8] Ding L, Getz G, Wheeler DA, Mardis ER, McLellan MD (2008). Somatic mutations affect key pathways in lung adenocarcinoma.. Nature.

[9] Meuwissen R, Berns A (2005). Mouse models for human lung cancer.. Genes Dev.

[10] Dankort D, Filenova E, Collado M, Serrano M, Jones K (2007). A new mouse model to explore the initiation, progression, and therapy of BRAFV600E-induced lung tumors.. Genes Dev.

[11] Perl AK, Wert SE, Loudy DE, Shan Z, Blair PA (2005). Conditional recombination reveals distinct subsets of epithelial cells in trachea, bronchi, and alveoli.. Am J Respir Cell Mol Biol.

[12] Ji H, Wang Z, Perera SA, Li D, Liang MC (2007). Mutations in BRAF and KRAS converge on activation of the mitogen-activated protein kinase pathway in lung cancer mouse models.. Cancer Res.

[13] Kerkhoff E, Fedorov LM, Siefken R, Walter AO, Papadopoulos T (2000). Lung-targeted expression of the c-Raf-1 kinase in transgenic mice exposes a novel oncogenic character of the wild-type protein.. Cell Growth Differ.

[14] Rice WR, Conkright JJ, Na CL, Ikegami M, Shannon JM (2002). Maintenance of the mouse type II cell phenotype in vitro.. Am J Physiol Lung Cell Mol Physiol.

[15] Rapp UR, Korn C, Ceteci F, Karreman C, Luetkenhaus K (2009). MYC is a metastasis gene for non-small-cell lung cancer.. PLoS One.

[16] Barnes PJ (2000). Mechanisms in COPD: differences from asthma.. Chest.

[17] Holgate ST, Davies DE, Lackie PM, Wilson SJ, Puddicombe SM (2000). Epithelial-mesenchymal interactions in the pathogenesis of asthma.. J Allergy Clin Immunol.

[18] Flanders KC (2004). Smad3 as a mediator of the fibrotic response.. Int J Exp Pathol.

[19] Willis BC, Borok Z (2007). TGF-beta-induced EMT: mechanisms and implications for fibrotic lung disease.. Am J Physiol Lung Cell Mol Physiol.

[20] Lee G, Walser TC, Dubinett SM (2009). Chronic inflammation, chronic obstructive pulmonary disease, and lung cancer.. Curr Opin Pulm Med.

[21] Yildirim AO, Muyal V, John G, Muller B, Seifart C (2010). Palifermin induces alveolar maintenance programs in emphysematous mice.. Am J Respir Crit Care Med.

[22] Mucenski ML, Wert SE, Nation JM, Loudy DE, Huelsken J (2003). beta-Catenin is required for specification of proximal/distal cell fate during lung morphogenesis.. J Biol Chem.

[23] Qu P, Roberts J, Li Y, Albrecht M, Cummings OW (2009). Stat3 downstream genes serve as biomarkers in human lung carcinomas and chronic obstructive pulmonary disease.. Lung Cancer.

[24] Mombaerts P, Iacomini J, Johnson RS, Herrup K, Tonegawa S (1992). RAG-1-deficient mice have no mature B and T lymphocytes.. Cell.

[25] Jarnicki A, Putoczki T, Ernst M (2010). Stat3: linking inflammation to epithelial cancer - more than a “gut” feeling?. Cell Div.

[26] de Torres JP, Bastarrika G, Wisnivesky JP, Alcaide AB, Campo A (2007). Assessing the relationship between lung cancer risk and emphysema detected on low-dose CT of the chest.. Chest.

[27] Wilson DO, Weissfeld JL, Balkan A, Schragin JG, Fuhrman CR (2008). Association of radiographic emphysema and airflow obstruction with lung cancer.. Am J Respir Crit Care Med.

[28] Schubbert S, Bollag G, Shannon K (2007). Deregulated Ras signaling in developmental disorders: new tricks for an old dog.. Curr Opin Genet Dev.

[29] Michaloglou C, Vredeveld LC, Soengas MS, Denoyelle C, Kuilman T (2005). BRAFE600-associated senescence-like cell cycle arrest of human naevi.. Nature.

[30] Shaw AT, Meissner A, Dowdle JA, Crowley D, Magendantz M (2007). Sprouty-2 regulates oncogenic K-ras in lung development and tumorigenesis.. Genes Dev.

[31] Curtis JL, Freeman CM, Hogg JC (2007). The immunopathogenesis of chronic obstructive pulmonary disease: insights from recent research.. Proc Am Thorac Soc.

[32] Li Y, Du H, Qin Y, Roberts J, Cummings OW (2007). Activation of the signal transducers and activators of the transcription 3 pathway in alveolar epithelial cells induces inflammation and adenocarcinomas in mouse lung.. Cancer Res.

[33] Sullivan NJ, Sasser AK, Axel AE, Vesuna F, Raman V (2009). Interleukin-6 induces an epithelial-mesenchymal transition phenotype in human breast cancer cells.. Oncogene.

[34] Andersen MH, Fensterle J, Ugurel S, Reker S, Houben R (2004). Immunogenicity of constitutively active V599EBRaf.. Cancer Res.

[35] Andersson CK, Mori M, Bjermer L, Lofdahl CG, Erjefalt JS (2010). Alterations in lung mast cell populations in patients with chronic obstructive pulmonary disease.. Am J Respir Crit Care Med.

[36] Krupke DM, Begley DA, Sundberg JP, Bult CJ, Eppig JT (2008). The Mouse Tumor Biology database.. Nat Rev Cancer.

