# Supplementary figures and images for: Expression of B-RAF V600E in Type II Pneumocytes Causes Abnormalities in Alveolar Formation, Airspace Enlargement and Tumor Formation in Mice
Source: PLoS One. 2011 Dec 14;6(12):e29093. doi: 10.1371/journal.pone.0029093 (PMC3237599; doi:10.1371/journal.pone.0029093)

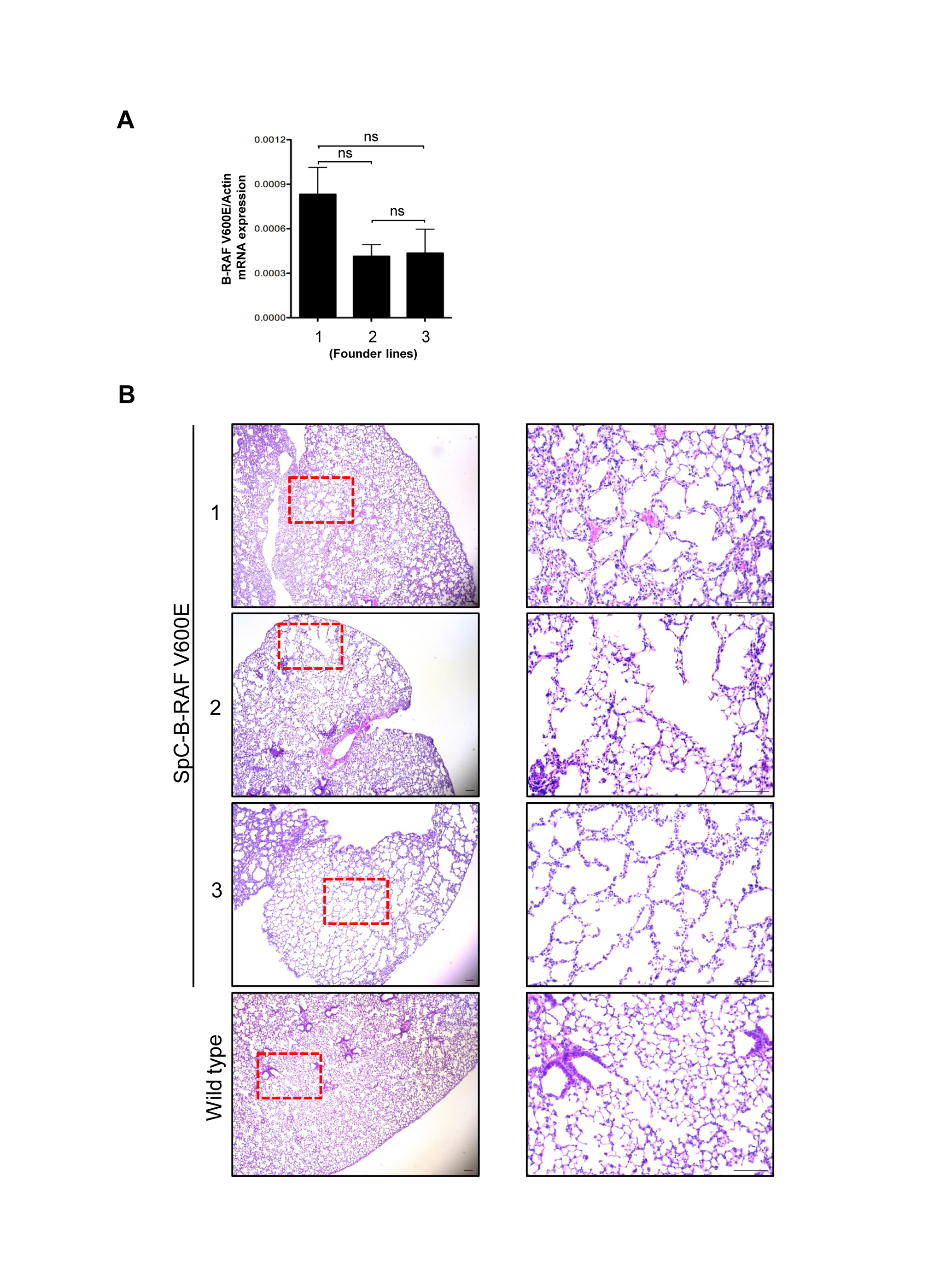

Supplement: Figure S1 — Comparable formation of airspace enlargements in different SpC-B-RAF V600E transgenic founder lines. (A) Analysis of B-RAF V600E mRNA levels between the founders (2 weeks old) by Real-Time PCR, data represent mean+SEM, (t-test ns = not significant, n = 3). (B) Representative paraffin embedded H&E stained lung sections from wild type and transgenic mice (2 months old), right panel pictures are the high magnification of red insets, scale bar = 100 µm. (TIF) [file pone.0029093.s001.tif]

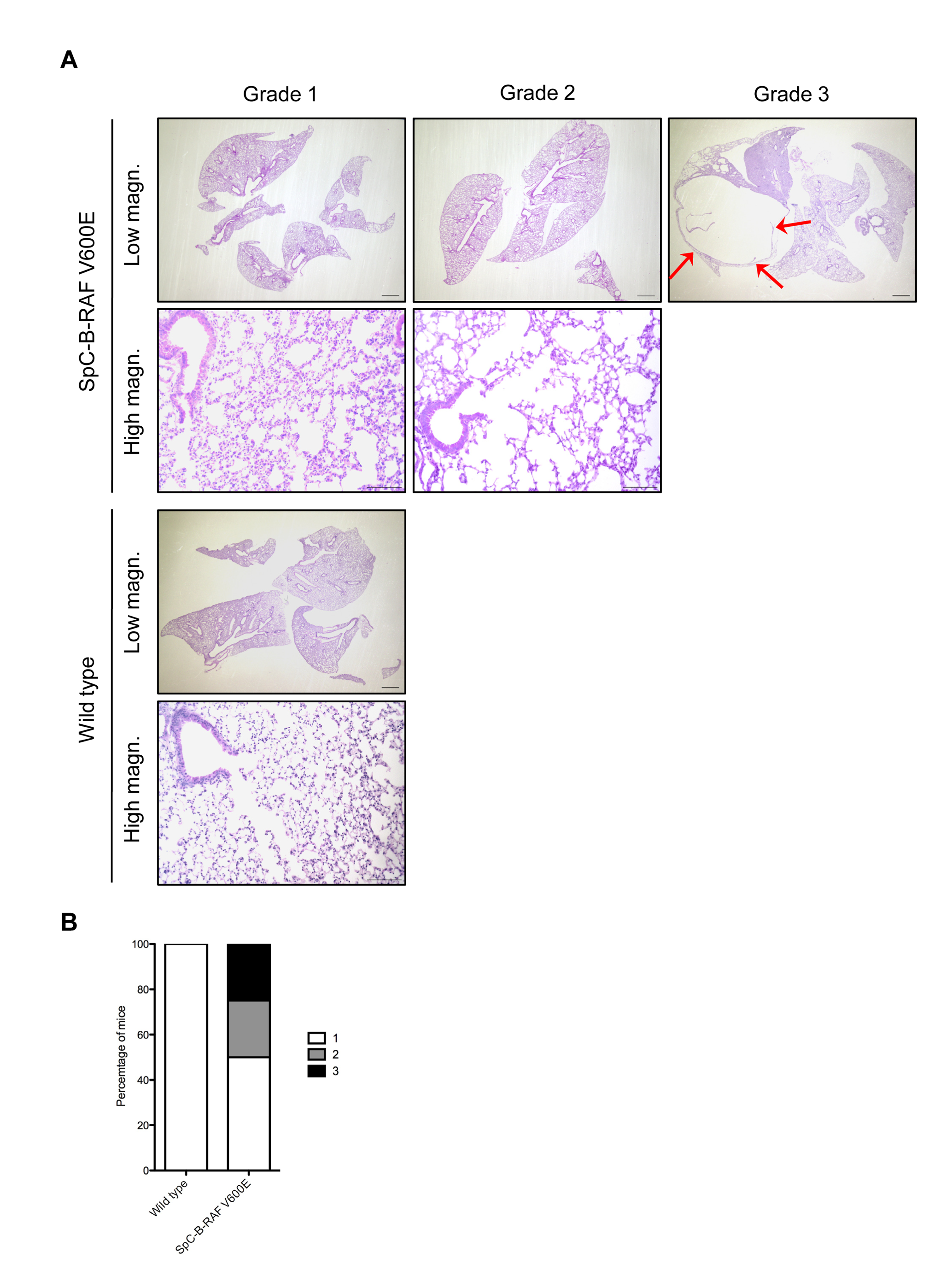

Supplement: Figure S2 — Scoring of B-RAF V600E induced lung lesions in transgenic mice. (A) Representative pictures of H&E stained lung sections from wild type and transgenic mice show airspace enlargements with different grades. The scoring was performed as follow: 1) Micro- and macro-scopically normal; 2) Macroscopically normal, but microscopically abnormal [airspace enlargement]; 3) Macroscopic abnormalities [bulb formation, pointed by arrows]. Scale bar = 1 mm for low magn. and 100 µm for high magn. pictures. (B) Incidence of the lung lesions with different grades, (n = 59 for wild type, n = 102 for SpC-B-RAF V600E). (TIF) [file pone.0029093.s002.tif]

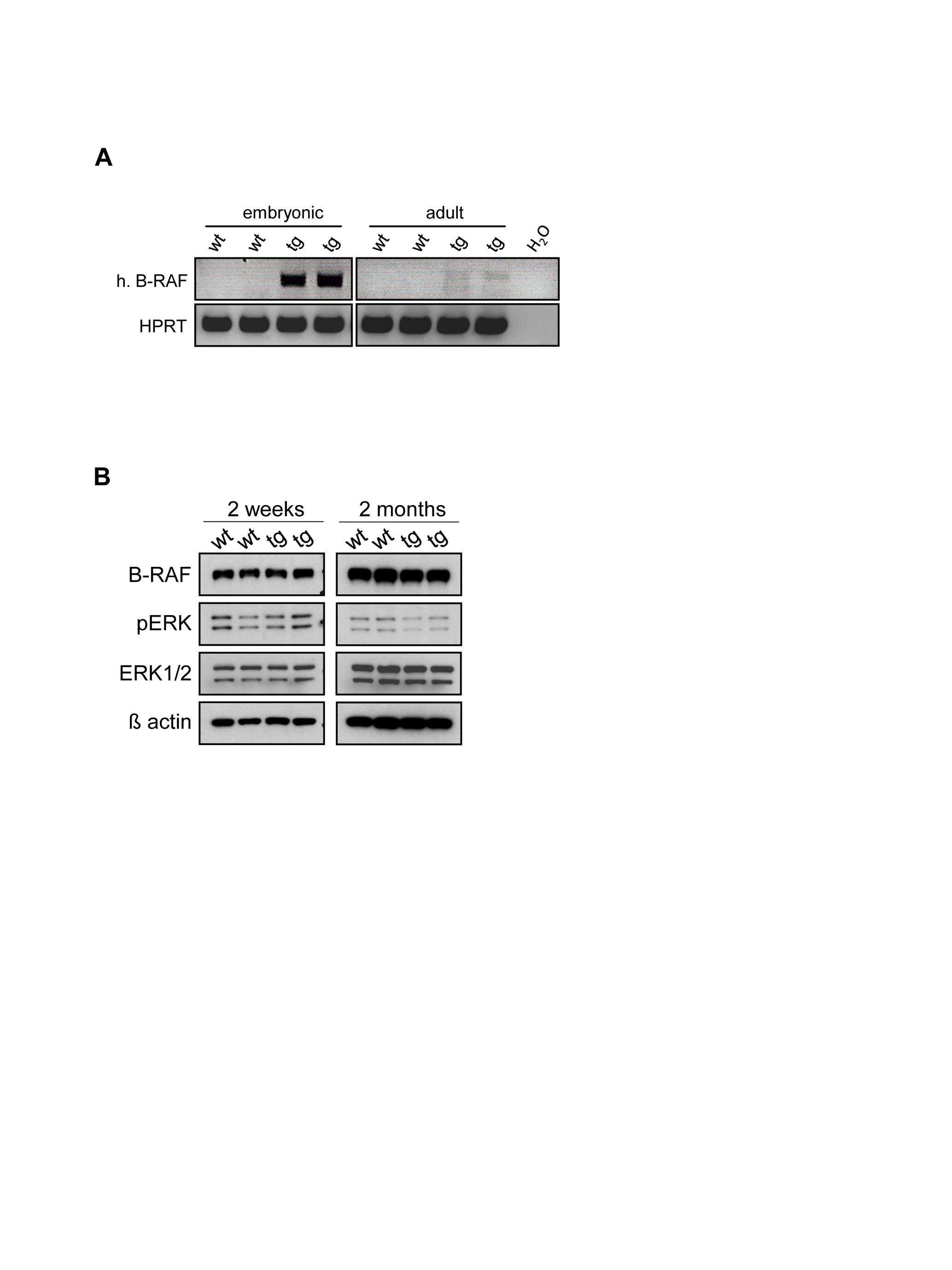

Supplement: Figure S3 — Diminished transgene expression and lack of phospho-ERK upregulation in SpC-B-RAF V600E mice. (A) Semi quantitative RT-PCR analysis of total lung RNA samples from wild type (wt) and transgenic (tg) animals shows different levels of transgene expression between embryonic and adult mice, HPRT was used as an internal control. (B) Total lung protein lysates from two wild type (wt) and transgenic (tg) littermates were gel separated and immunoblotted; ages and antibodies are as indicated. (TIF) [file pone.0029093.s003.tif]

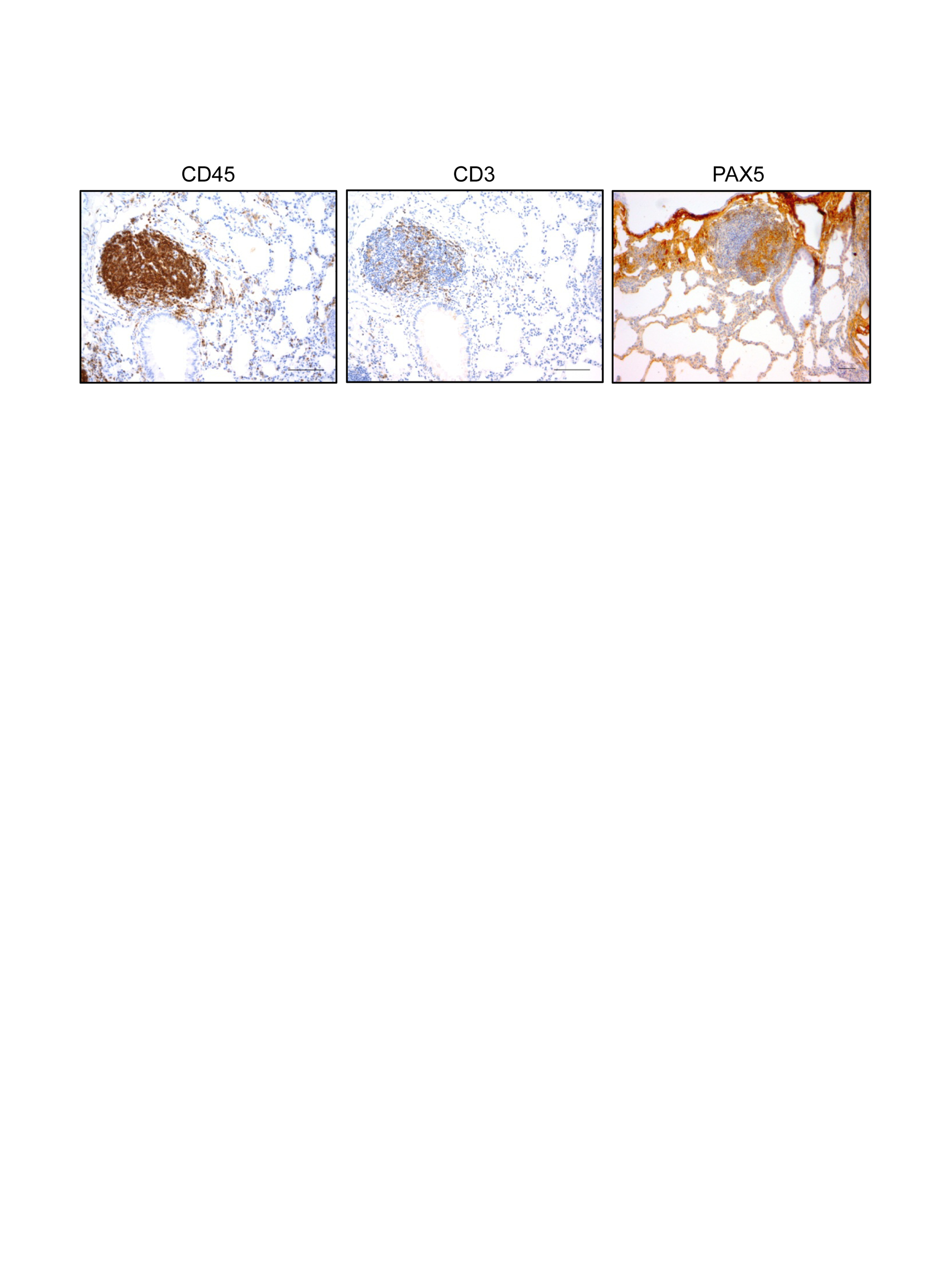

Supplement: Figure S4 — Presence of T- and B- cells in CD45 positive clusters. Lung sections from SpC-B-RAF V600E transgenic mice were stained for CD3 and PAX5 to identify T- and B- cells, respectively; hematoxylin was used as a counterstain; scale bar = 100 µm. (TIF) [file pone.0029093.s004.tif]

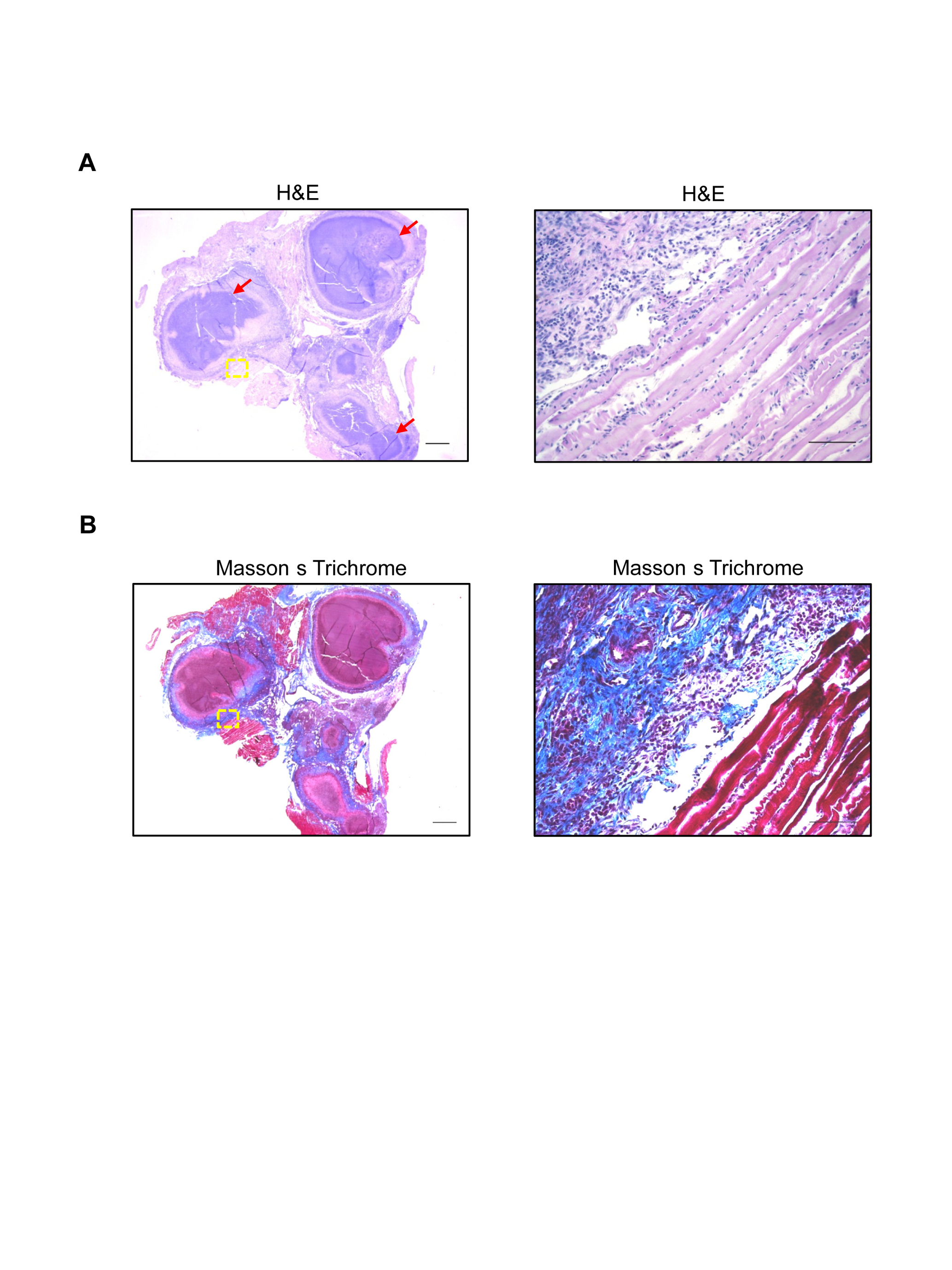

Supplement: Figure S5 — Severe lung degeneration in an aged (17.5 months) mouse transgenic for B-RAF V600E. (A) H&E staining of a lung section shows massive infiltration of leukocytes (red arrows). (B) Masson's Trichrome staining of the consecutive lung section reveals collagen-enriched regions (blue), the right panel are high magnifications of the yellow inserts; scale bar = 1 mm for the left and 100 µm for the right panel. (TIF) [file pone.0029093.s005.tif]

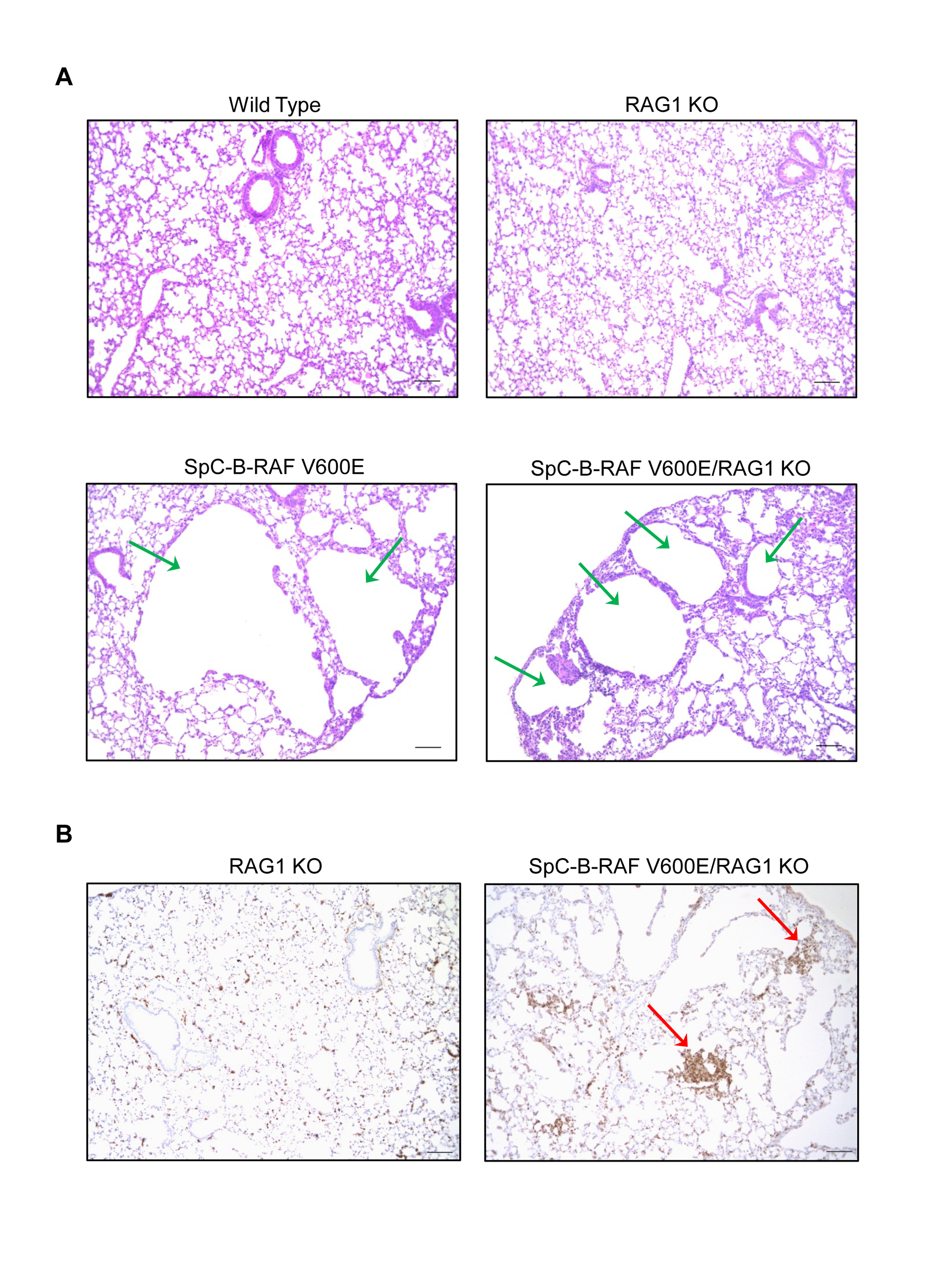

Supplement: Figure S6 — Mature T- and B- cells are dispensable for the formation of SpC-B-RAF V600E-induced lung lesions. (A) H&E staining of lung sections from wild type, RAG1 knock out (KO), SpC-B-RAF V600E single transgenic and SpC-B-RAF V600E/RAG1 KO compound animals show the persistence of airspace enlargements in SpC-B-RAF V600E single and compound mice, green arrows point to airspace enlargements. (B) Immunostaining of paraffin embedded lung sections from RAG1 KO single transgenic and SpC-B-RAF V600E/RAG1 KO compound animals for CD45 (brown), red arrows point to CD45 positive clusters, hematoxylin was used as a counterstain; scale bar = 100 µm. (TIF) [file pone.0029093.s006.tif]

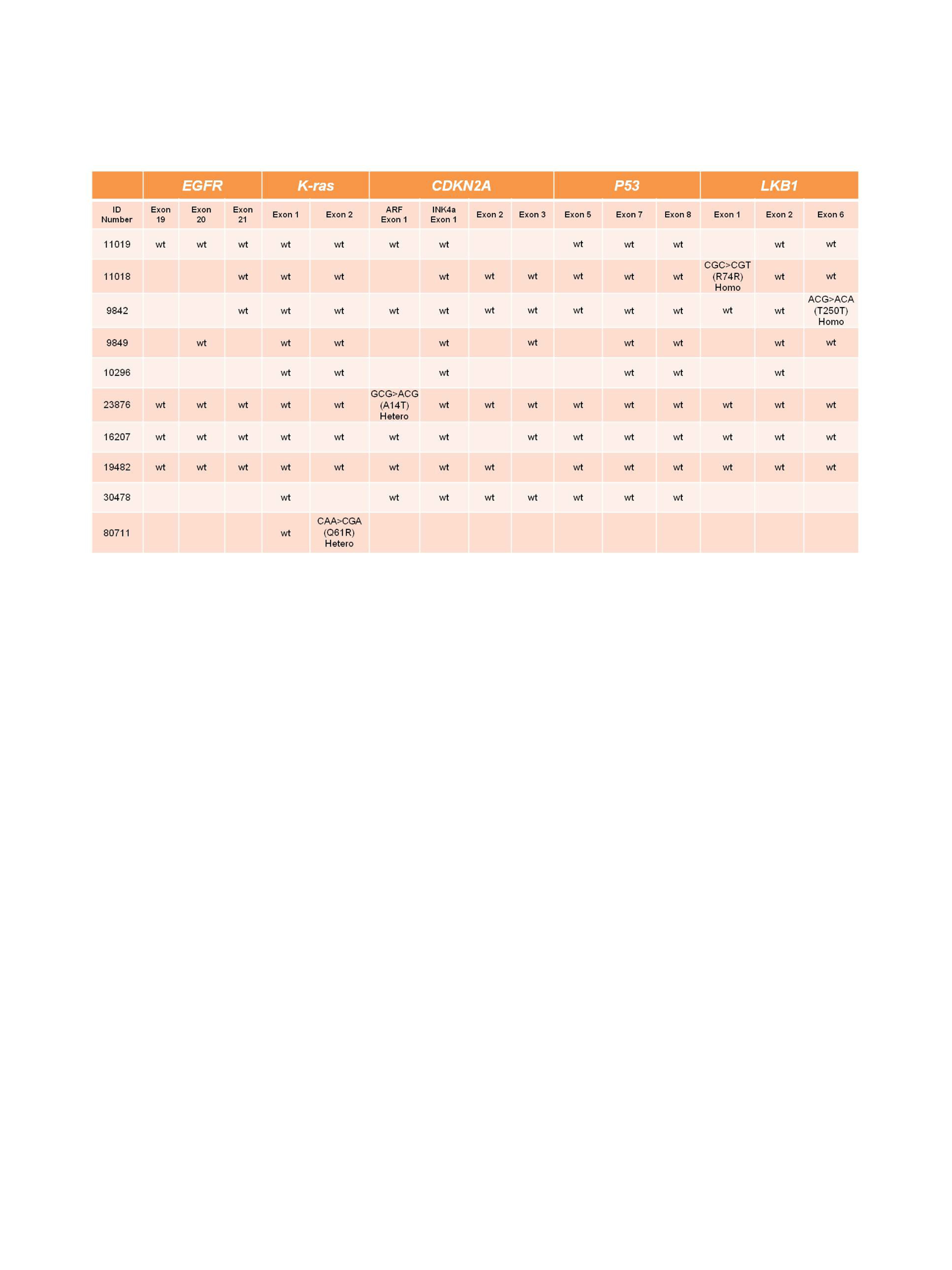

Supplement: Figure S7 — Lung tumors found in SpC-B-RAF V600E transgenic mice in general do not harbor mutations frequently present in human and mouse NSCLC. Genomic DNA from lung tumors of SpC-B-RAF V600E transgenic mice were screened for the indicated genes, individual animal (ID) and Exon numbers are as indicated, wt = wild type, Hetero = heterozygous, Homo = homozygous, empty boxes represent untested samples. (TIF) [file pone.0029093.s007.tif]
