# Supplementary material for: Expression of B-RAF V600E in Type II Pneumocytes Causes Abnormalities in Alveolar Formation, Airspace Enlargement and Tumor Formation in Mice
Source: PLoS One. 2011 Dec 14;6(12):e29093. doi: 10.1371/journal.pone.0029093 (PMC3237599; doi:10.1371/journal.pone.0029093)
Supplement: Table S1 — Primers for Real time PCR. (DOCX) [file pone.0029093.s008.docx]

| Gene | Forward primer | Reverse primer |
| --- | --- | --- |
| ***E-Cadherin*** | GCTGGACCGAGAGAGTTA | TCGTTCTCCACTCTCACAT |
| ***Mouse-HPRT*** | TCAGTCAACGGGGGACATAAA | GGGGCTGTACTGCTTAACCAG |
| ***Human B-RAF (V600E)*** | GTCATCTTCATCCTCAGAAG | TTCAACATTTTCACTGCCAC |
| ***Mouse B-RAF*** | CATCTTCTTCCTCATCCTCG | TTCAACATTTTCACTGCCAC |
| ***Mouse Vimentin*** | GTGCGCCAGCAGTATGAAAG | GCATCGTTGTTCCGGTTGG |
| ***ß actin*** | GTCGTACCACAGGCATTGTGATGG | GCAATGCCTGGGTACATGGTGG |

**Table S1. Primers for Real time PCR**
