# Supplementary material for: Expression of B-RAF V600E in Type II Pneumocytes Causes Abnormalities in Alveolar Formation, Airspace Enlargement and Tumor Formation in Mice
Source: PLoS One. 2011 Dec 14;6(12):e29093. doi: 10.1371/journal.pone.0029093 (PMC3237599; doi:10.1371/journal.pone.0029093)
Supplement: Table S2 — Primers for the amplification of genomic DNA. (DOCX) [file pone.0029093.s009.docx]

**Table S2. Primers for the amplification of genomic DNA**

| Gene | Forward primer | Reverse primer |
| --- | --- | --- |
| ***EGFR* exon 19** | CCAGCTCACAAGGCAACATG | CCCACGTCCCTATAAGCAGA |
| ***EGFR* exon 20** | AAGGGATATGCGTGCCTCTC | GGGTACTTCAGTGGACAGAC |
| ***EGFR* exon 21** | ACCCTGTGTTCAGGTGCATG | CTGGGCTGTCAGGAAAATGC |
| ***K-RAS* exon 1** | ATGACTGAGTATAAACTTGT | TCGTACTCATCCTCAAAGTG |
| ***K-RAS* exon 2** | TACAGGAAACAAGTAGTAATTGATGGAGAA | ATAATGGTGAATATCTTCAAATGATTTAGT |
| ***p19^ARF^ exon 1*** | TTGTCACAGTGAGGCCGCCG | GCACCTGTGCGTGCAGCTTC |
| ***p16^INK4a^* exon 1** | GCGAACTCGAGGAGAGCCAT | TTACCCGACTGCAGATGGGACAC |
| ***p16^INK4a^* exon 2** | CTGGCCGTGATCCCTCTACT | GAGTGTCAGAAGCTTTTGGACCAA |
| ***p16^INK4a^* exon 3** | CTGGCACCTAGGACAGCTTTATA | GAAAAAGGCGGGCTGAGGCC |
| ***p53* exon 5** | GCTTGTCCCCGACCTCCGTT | GGCTGCCAGTCCTAACCCCAC |
| ***p5*3 exon 7** | CCACAGGTCTCCCCAAGG | TGGCAAGTGGCTCCTGAC |
| ***p5*3 exon 8** | TCTTACTGCCTTGTGCTGGTCC | AGGCTCCTCCGCCTCCTT |
| ***LKB1* exon 1** | GCTCCCGAAGGGGACGAGGA | AGGAAGGGCTGCCCAAGGAG |
| ***LKB1* exon 2** | CAGCAGGCAGGGTCAGCTAA | CCAGCCCACTCCACCCACAG |
| ***LKB1* exon 6** | ACCCTGTAGCGGGGGG | CCTCCCATCCGGACAA |
